# Structural and Functional Characterization of a Single-Chain Form of the Recognition Domain of Complement Protein C1q

**DOI:** 10.3389/fimmu.2016.00079

**Published:** 2016-03-02

**Authors:** Christophe Moreau, Isabelle Bally, Anne Chouquet, Barbara Bottazzi, Berhane Ghebrehiwet, Christine Gaboriaud, Nicole Thielens

**Affiliations:** ^1^IBS, University of Grenoble Alpes, Grenoble, France; ^2^CNRS, IBS, Grenoble, France; ^3^IBS, CEA, Grenoble, France; ^4^Immunopharmacology Laboratory, Humanitas Research Hospital, Rozzano, Italy; ^5^Department of Medicine, Stony Brook University, Stony Brook, NY, USA

**Keywords:** innate immune recognition, complement, C1q, protein engineering, X-ray crystallography, surface plasmon resonance

## Abstract

Complement C1q is a soluble pattern recognition molecule comprising six heterotrimeric subunits assembled from three polypeptide chains (A–C). Each heterotrimer forms a collagen-like stem prolonged by a globular recognition domain. These recognition domains sense a wide variety of ligands, including pathogens and altered-self components. Ligand recognition is either direct or mediated by immunoglobulins or pentraxins. Multivalent binding of C1q to its targets triggers immune effector mechanisms mediated *via* its collagen-like stems. The induced immune response includes activation of the classical complement pathway and enhancement of the phagocytosis of the recognized target. We report here, the first production of a single-chain recombinant form of human C1q globular region (C1q-scGR). The three monomers have been linked in tandem to generate a single continuous polypeptide, based on a strategy previously used for adiponectin, a protein structurally related to C1q. The resulting C1q-scGR protein was produced at high yield in stably transfected 293-F mammalian cells. Recombinant C1q-scGR was correctly folded, as demonstrated by its X-ray crystal structure solved at a resolution of 1.35 Å. Its interaction properties were assessed by surface plasmon resonance analysis using the following physiological C1q ligands: the receptor for C1q globular heads, the long pentraxin PTX3, calreticulin, and heparin. The 3D structure and the binding properties of C1q-scGR were similar to those of the three-chain fragment generated by collagenase digestion of serum-derived C1q. Comparison of the interaction properties of the fragments with those of native C1q provided insights into the avidity component associated with the hexameric assembly of C1q. The interest of this functional recombinant form of the recognition domains of C1q in basic research and its potential biomedical applications are discussed.

## Introduction

The soluble defense collagens are oligomeric innate immune pattern recognition receptors (PRRs), which are composed of N-terminal collagen-like stems prolonged by C-terminal globular trimeric pattern recognition domains [reviewed in Ref. (1)]. According to the nature of their recognition domain, these PRRs can be divided into three families in human, namely, proteins with gC1q domains (C1q and adiponectin), C-type lectin carbohydrate recognition domains (lung surfactant proteins A and D, mannan-binding lectin (MBL), collectins kidney 1 and liver 1), and fibrinogen-like domains (ficolins). Following target recognition, C1q and collectins (except surfactant proteins) or ficolins have the capacity to trigger activation of the classical and lectin pathways of complement for microbial killing and phagocytosis, through proteases associated with their collagen stems.

The C1q molecule is a complex defense collagen, being assembled from six heterotrimeric subunits, each consisting of three homologous, yet distinct polypeptide chains (A–C) encoded by the *C1QA*, *C1QB*, and *C1QC* genes oriented in the A–C–B order on human chromosome 1p (2). C1q also features the most versatile recognition properties, being able to identify not only bacterial and viral pathogens, either directly or through other immune proteins such as antibodies and pentraxins, but also many altered self elements, including β-amyloid fibrils (3), the pathological form of the prion protein (4, 5), modified low-density lipoproteins (6), and apoptotic cells (7–9).

Production of the C1q globular region (C1q-GR) by limited proteolysis of the serum-derived protein with collagenase allowed resolution of its X-ray crystal structure. The resulting compact heterotrimeric structure revealed differences in the surface charges of the subunits, a key factor for the versatility of C1q binding properties (10, 11). A further step toward understanding C1q binding properties was accomplished with the production of recombinant forms of the individual gC1q domains fused to maltose-binding protein, which revealed that these domains are functionally autonomous modules with differential ligand-­binding properties (12). Site-directed mutagenesis studies provided information about the residues involved in the interaction of C1q with some of its ligands (13–16). However, elucidation of the C1q recognition properties in the more physiological context of the heterotrimeric globular regions still awaits the availability of the corresponding recombinant fragment.

We report here, the production of a single-chain recombinant form of human C1q globular region (C1q-scGR). The three monomers have been linked in tandem to generate a single continuous polypeptide, based on a strategy previously used to generate a single-chain form of the homotrimeric globular domain of adiponectin, a protein structurally related to C1q (17). The C1q-scGR recombinant protein was produced at high yield in stably transfected mammalian cells. Its physicochemical, structural, and functional analysis shows that it is correctly folded and retains the ability to associate with physiological C1q ligands, including the long pentraxin PTX3, the receptor for the globular heads of C1q (gC1qR), calreticulin (CRT), and heparin. The interest of this fragment in basic research and its potential biomedical applications will be discussed.

## Materials and Methods

### Proteins and Reagents

C1q was purified from human serum and quantified, as described previously (18). The globular regions of C1q were prepared by collagenase digestion of C1q, as described previously (3), and their molar concentration estimated using a Mw value of 48,000 and an absorption coefficient (A1%, 1 cm) at 280 nm of 0.93. Recombinant human PTX3, gC1qR, and CRT were produced, as described previously (19–21). Streptavidin and heparin-biotin sodium salt (Mw 15 kDa) were procured from Sigma-Aldrich. Oligonucleotides were purchased from Eurogentec. Restriction and modification enzymes were from New England Biolabs.

### Cloning of the Single-Chain Globular Domain of Human C1q

For recombinant protein expression in the baculovirus/insect cells system, a synthetic cDNA encoding residues 85–223 of mature C1qA, a Gly–Ser–Gly linker, residues 87–217 of mature C1qC (gC1qC), a Gly–Ser–Ala linker, and residues 90–226 of mature C1qB (gC1qB), cloned in frame with the melittin signal peptide of the pNT-Bac vector (22) (pNT-Bac–C1q-scGR), was purchased from GeneCust.

For expression in mammalian 293-F cells, an intermediate construct was generated from the pcDNA3.1–C1qA vector (23) by removing residues 1–87 of mature C1qA by site-directed mutagenesis, allowing in frame cloning of residues 88–223 of C1qA with the native signal peptide of C1qA (pcDNA3.1–gC1qA). A DNA fragment encoding the Gly–Ser–Gly linker, gC1qC, the Gly–Ser–Ala linker, and gC1qB was amplified using VentR polymerase and pNT-Bac–C1q-scGR as a template and inserted into pcDNA3.1–gC1qA by site-directed mutagenesis. The resulting construct (called pcDNA3.1–C1q-scGR) was characterized by restriction mapping and checked by double-stranded DNA sequencing (GATC Biotech).

### Production of C1q-scGR in Eukaryotic Cells and Protein Purification

Generation of a recombinant baculovirus from the pNT-Bac–C1q-scGR plasmid using the Bac-to-Bac system (Invitrogen) and infection of *Trichoplusia ni* (High Five) insect cells was performed, as described previously (24). Stably transfected cells producing C1q-scGR were obtained by transfection of FreeStyle 293-F cells with the pcDNA3.1–C1q-scGR plasmid using 293-fectin and subsequent selection with 400 μg/ml G418 as recommended by the manufacturer (Invitrogen). The cells were expanded in the Freestyle expression medium (Invitrogen) and the culture supernatant harvested and replaced every 72 h up to three times.

The insect and mammalian cell culture supernatants containing C1q-scGR (500 ml) were dialyzed against 50 mM MES, 25 mM NaCl, pH 6.4, and loaded at 1.5 ml/min onto a SP Sepharose Fast Flow column (GE Healthcare) (50 ml) equilibrated in the same buffer. Elution was carried out by applying a 1-l linear gradient from 25 to 500 mM NaCl in the same buffer. The fractions containing the recombinant protein were identified by SDS-PAGE analysis, pooled, dialyzed against 50 mM Tris–HCl, 150 mM NaCl, pH 7.4, and concentrated to 1–5 mg/ml by ultrafiltration on a PM-10 membrane (Amicon). The molar concentration of C1q-scGR was estimated using an absorption coefficient (A1%, 1 cm) at 280 nm of 0.93 and a Mr value of 47,534, as determined by mass spectrometry.

### SDS-PAGE, N-Terminal Sequence, and Liquid Chromatography–Electrospray Ionization–Time-of-Flight Mass Spectrometry Analyses

Recombinant C1q-scGR was analyzed by SDS-PAGE under non-reducing or reducing conditions using Tris–HCl gels containing 10% polyacrylamide. N-terminal sequence determination was performed using an Applied Biosystems gas-phase sequencer model 492 coupled online with an Applied Biosystems Model 140C HPLC system. Liquid chromatography–electrospray ionization–time-of-flight (LC–ESI–TOF) mass spectrometry analyses of purified C1q-scGR, before and after treatment with *Clostridium perfringens* type X neuraminidase (Sigma) (0.3 U/mg) for 5 h at 25°C, were performed using a 6210 LC–TOF mass spectrometer interfaced with LC pump system (Agilent Technologies). Samples were desalted on-line on a protein trap (Zorbax 300SB-C8, 5 μm, 5 mm × 0.3 mm, Agilent Technologies) before analysis.

### Analytical Ultracentrifugation

Sedimentation velocity analysis was performed using a Beckman XL-I analytical ultracentrifuge and an AN-50 TI rotor (Beckman Coulter, Palo Alto, CA, USA). Three C1q-scGR samples at 0.9, 2.5, and 5 mg/ml were loaded into 12, 3, and 1.5 mm pathlength double-sector cells and centrifuged at 42,000 rpm at 6°C in 50 mM Tris–HCl, 150 mM NaCl, and pH 7.4. Data acquisition was done in absorbance (at 280 nm) and interference modes. The sedimentation coefficients were obtained by fitting the sedimentation velocity profiles to the non-interacting species model using the SEDFIT program,^1^ and the continuous distribution of sedimentation coefficients was obtained considering globular proteins. Solvent density was calculated at 1.00739 g/ml, and the partial specific volume was estimated at 0.724 ml/g, using the SEDNTERP program.^2^

### Crystallization, Data Collection, and Structure Determination

Single-chain recombinant form of human C1q globular region was concentrated to 5 mg/ml, and standard crystallization kits were screened through the EMBL HTX Lab platform at 20°C. Several initial hits were reproduced manually, using the hanging drop method by mixing equal volumes (2 μl) of the protein and reservoir solutions and adding calcium in some reservoir solutions. The crystallization conditions used and the resulting crystal morphology were very similar to those obtained previously for plasma-derived C1q-GR (10). To obtain the crystal structures presented here, the following reservoir solutions were used: (1) 30% PEG 8000, 0.1M Hepes, pH 7.5; (2) 23% PEG 3350, 0.1M Tris, 0.2M NaCl, 50 mM CaCl_2_, pH 8.5. Diffraction data were recorded up to 1.35 or 1.55 Å resolution at the European Synchrotron Radiation facility (ESRF) beamline ID23-eh1 and auto-processed in the C2 space group (25). The data collection statistics are provided in Table 1.

**Table 1:** **Data collection and refinement statistics for C1q-scGR**.

Reservoir solution PDB ID	Without calcium 5HZF	With calcium 5HKJ
**Data collection statistics**		
Unit cell lengths (Å)	81.0, 52.9, 89.9	81.1, 52.7, 89.9
Unit cell angles (°)	90, 115.2, 90	90, 115.2, 90
Resolution (Å)^a^	100.0–1.55 (1.61–1.55)	100.0–1.35 (1.4–1.35)
Rsym^a^	5.6 (75.3)	7.0 (67.1)
% completeness^a^	98.4 (96.7)	98.7 (94.7)
I/sigma (I) average^a^	14.8 (1.9)	11.3 (1.8)
No. of observed reflections^a^	243,827 (25,061)	363,221 (31,079)
No. of unique reflections^a^	49,348 (5211)	74,618 (7346)
CC ½^a^	99.9 (56)	99.8 (68.2)
Mean Wilson B	27	21
**Model refinement statistics**		
*R*_work_	0.169	0.175
*R*_free_	0.185	0.2021
Root mean square deviation bonds (Å)	0.011	0.016
Root mean square deviation angles (°)	1.19	1.64

*^a^Statistics for the high-resolution bin are in parentheses*.

The position and orientation of the C1q-scGR trimeric globular domain in the asymmetric unit were determined with the molecular replacement software Phaser (26). Alternative cycles of refinement and graphics edition were performed using Refmac5 (27) and Coot (28), respectively. The final refinement cycles were performed using Phenix (29). Refinement statistics are provided in Table 1. Illustrations were prepared using Pymol (30).

### Surface Plasmon Resonance Studies

Analyses were performed at 25°C using a Biacore 3000 instrument (GE Healthcare).

#### SPR Analyses on Immobilized C1q Protein Ligands

Calreticulin, gC1qR, and PTX3 were diluted to 20, 68, and 100 μg/ml in 10 mM sodium acetate pH 4.0, 4.0, and 3.5, respectively, and immobilized on a CM5 sensor chip (GE Healthcare) using the amine coupling chemistry in 10 mM Hepes, 150 mM NaCl, 3 mM EDTA, 0.005% surfactant P20, pH 7.4. The reference surface was submitted to the coupling steps without immobilized protein. Binding of C1q-scGR, C1q-GR, and C1q to immobilized CRT (3000–4700 RU), gC1qR (500–3400 RU), and PTX3 (5300–6200 RU) was measured at a flow rate of 20 μl/min in 50 mM Tris–HCl, 150 mM NaCl, 2 mM CaCl_2,_ 0.005% surfactant P20, pH 7.4. The specific binding signal was obtained by subtracting the background signal over the reference surface. Regeneration of the surfaces was achieved by 10 μl injections of 10–20 mM NaOH.

#### SPR Analyses on Immobilized Heparin

Streptavidin (approximately 4000 RU) was immobilized on two flow cells of a CM5 sensor chip, as described previously (31). Biotinylated heparin was captured on the streptavidin surface in 10 mM Hepes, 150 mM NaCl, 0.005% surfactant P20, pH 7.4 (HBS-P) until a coupling level of 250–300 RU was obtained. Serum C1q-GR and recombinant C1q-scGR were injected over the heparin-bound surface at 20 μl/min in HBS-P. Surfaces were regenerated with 10 μl of 1M NaCl. The streptavidin surface without bound heparin was used as a reference.

#### SPR Data Evaluation

Data were analyzed by global fitting either to a 1:1 Langmuir binding model or to a two-state reaction binding model of both the association and dissociation phases for at least five concentrations simultaneously, using the BIAevaluation 3.2 software (GE Healthcare). Buffer blanks were subtracted from the data sets used for kinetic analysis (double referencing). Chi^2^ values were below 3.5 in all cases. For the two-state reaction (conformational change) model, the apparent dissociation constants were calculated from the rate constants: *K*_D_ = 1/[(*k*_a1_/*k*_d1_) (1 + *k*_a2_/*k*_d2_)]. For the Langmuir binding model, the apparent equilibrium dissociation constants (*K*_D_) were calculated from the ratio of the dissociation and association rate constants (*k*_d_/*k*_a_).

## Results and Discussion

### Generation of a Single-Chain Recombinant Form of gC1q

**Figure 1:**
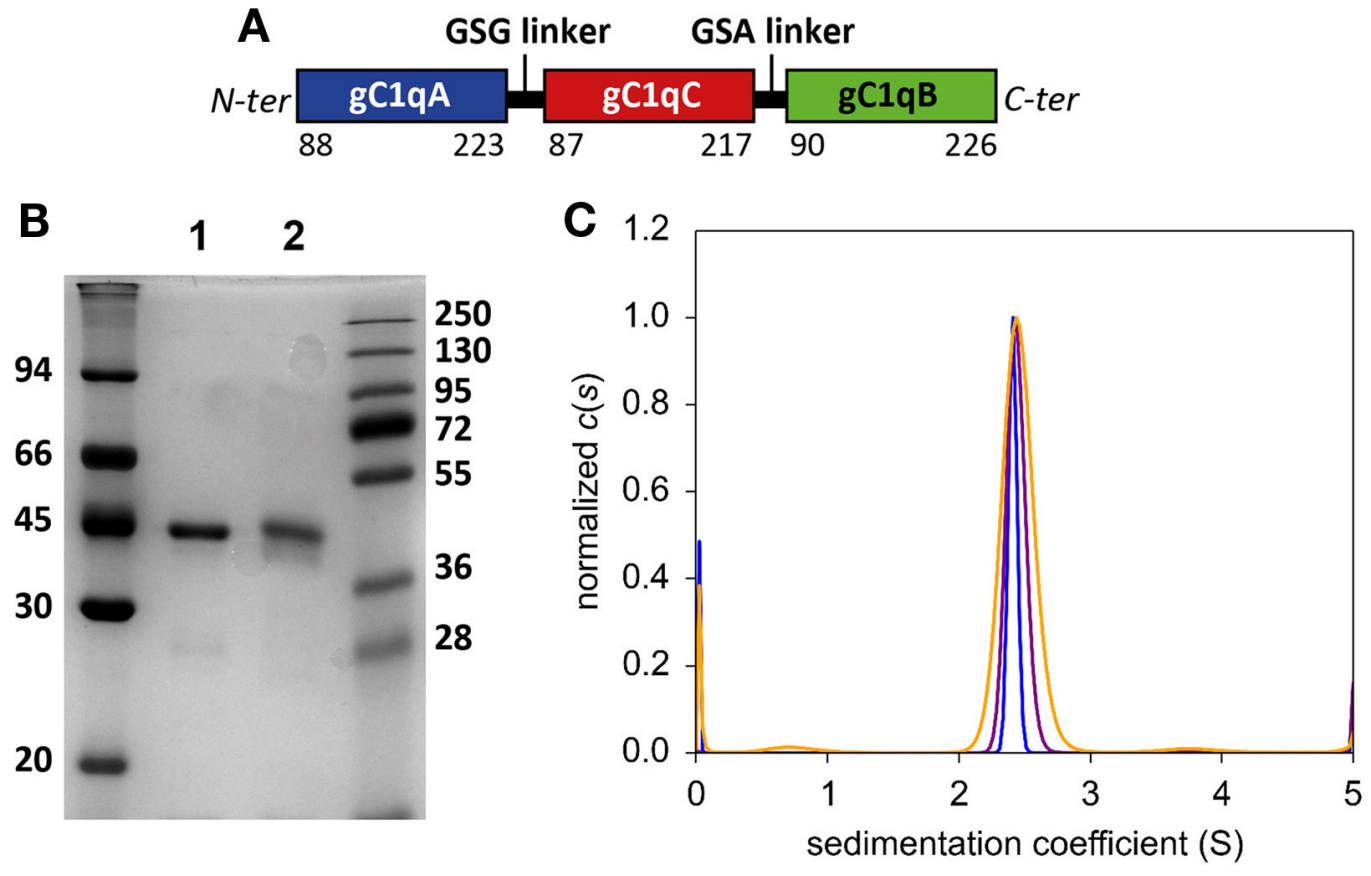
**Biochemical characterization of purified C1q-scGR**. **(A)** Schematic representation of mature recombinant C1q-scGR protein. **(B)** SDS-PAGE analysis of C1q-scGR, unreduced (lane 1) and reduced (lane 2). The molecular masses of unreduced and reduced markers (expressed in kilodaltons) are shown on the left and right sides of the gel, respectively. **(C)** Sedimentation velocity analysis of C1q-scGR. Analysis was performed as described in Section “Materials and Methods” and the continuous distribution of sedimentation coefficients is shown. Orange, purple and blue traces correspond to C1q-scGR samples at 0.9, 2.5, and 5 mg/ml, respectively (data acquisition in absorbance mode).

A strategy derived from that used for expression of a single polypeptide protein containing three consecutive copies of the globular domain of adiponectin was chosen to produce C1q-scGR (17). As revealed by the X-ray crystal structures of the globular domains of adiponectin (32) and of C1q (10), the N- and C-termini of the three gC1q modules emerge at the base of the trimer. Short 3-amino acid linkers can thus connect the adjacent monomers A–C and C–B. The 5′-3′ A–C–B order chosen to generate C1q-scGR also corresponds to that of the three C1q genes on chromosome 1p (33) (Figure 1A).

A first attempt to produce recombinant C1q-scGR was performed using a baculovirus/insect cells expression system, but the production yield was rather low since only 1 mg purified protein was recovered per liter of cell culture supernatant. In addition, the recombinant material was heterogeneous, consisting of a mixture of glycosylated and unglycosylated species (data not shown). Recombinant C1q-scGR was next produced in stably transfected 293-F mammalian cells and purified by cation-exchange chromatography. Up to 50 mg C1q-scGR could be purified from one liter of 293-F cells supernatant, which represents a 50-times higher yield compared to the baculovirus-infected insect cells. SDS-PAGE analysis showed a single band with an apparent mass of approximately 45 kDa under reducing and non-reducing conditions (Figure 1B).

N-terminal sequence analysis yielded the single sequence Lys–Asp–Gln–Pro–Arg, starting as expected at residue Lys 88 of C1qA chain. Mass spectrometry analysis yielded three peaks with masses of 47,749.84, 47,897.45, and 48,043.18 Da, accounting for a polypeptide chain with a predicted mass of 45,691.89 Da, and additional masses of 2059, 2205, and 2350 Da, corresponding to the three types of biantennary *N*-glycans (monosialylated, monosialylated fucosylated, and bisialylated) identified previously in serum-derived C1q (34, 35). The single N-glysosylation site at Asn 124 of C1qA is thus glycosylated in recombinant C1q-scGR. Sialidase treatment resulted in the appearance of two peaks with masses of 47,615.55 and 47,453.09 Da, compatible with asialylated biantennary *N*-glycans, fucosylated, or not (expected masses 1914 and 1768 Da).

Analysis of C1q-scGR by sedimentation velocity at three protein concentrations (0.9, 2.5, and 5 mg/ml) yielded a major peak accounting for 95 ± 3% of the signal with a sedimentation coefficient of 2.14 ± 0.4 S. Analysis in non-interacting species yielded a molecular mass of 41.4 ± 2 kDa, which is close to the mass measured by mass spectrometry, indicating that C1q-scGR is a monomer (Figure 1C).

### X-ray Crystal Structure of C1q-scGR

**Figure 2:**
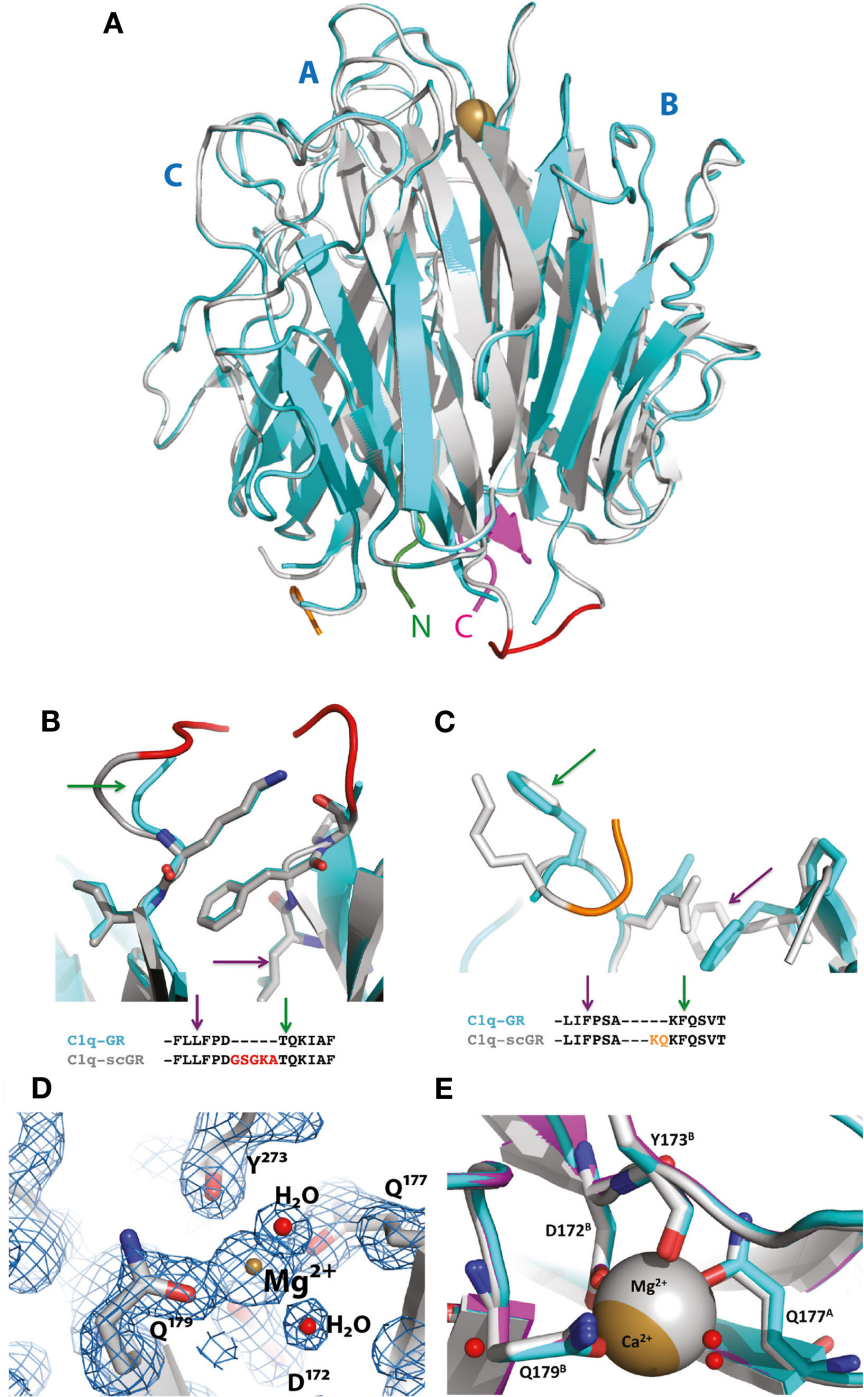
**Crystal structure of C1q-scGR and its comparison with C1q-GR**. **(A)** The global superposition of C1q-scGR (gray) and C1q-GR (cyan) structures illustrates their similarity, with only 0.1 Å RMS deviation on 331 common Cα positions. The A–C labels corresponding to the three C1q-GR subunits are shown in blue. The calcium ion is shown as a golden sphere. The N-terminal and C-terminal ends are shown in green and magenta, respectively. More details of the C–B (red) and A–C (orange) linkers are provided in **(B)** and **(C)**, respectively. The green and magenta arrows link the details in these zooming sections with the positions in the sequences shown below. **(D)** In the absence of calcium in the crystallization reservoir, the calcium-binding site is occupied by a magnesium ion, as illustrated here in the 2mFo–DFc map contoured at 2 σ level. Introducing a calcium ion in the model at this position deteriorates the *R*_free_ factor by 2% and introduces a negative peak at this position in the Fo–Fc electron density map (not shown). **(E)** The ion substitution does not alter the structure of the calcium-binding site environment, as illustrated by the superposition of the structures of C1q-GR (cyan), C1q-scGR with calcium (magenta), and C1q-scGR with magnesium (gray).

Although sialidase treatment of serum-derived C1q-GR had been required to obtain crystals of this protein suitable for structure determination (10), the presence of sialic acids in C1q-scGR was not an obstacle to the determination of its crystal structure. Its X-ray structure, refined at 1.35 Å resolution (Table 1), allowed us to check that the linkers did not introduce any distortion. The recombinant and serum-derived C1q globular domains are indeed almost identical, as shown by their very small 0.1 Å RMS deviation on the 331 Cα common positions (Figure 2A). The main-chain trace of the segment encompassing the C–B linker and the first two residues of gC1qB (GSAKA) were modeled into the electron density (Figure 2B). This more rigid C–B linker only slightly alters the main-chain position of the two preceding and following residues, but the positions of their side-chains are conserved (Figure 2B). The A–C linker is more flexible, and only the first two residues of gC1qC were modeled into the electron density (Figure 2C). All the water molecules except one correspond to those observed in the structure of the plasma-derived protein (PDB code 2wnv) (36). In the absence of calcium in the reservoir solution, the electron density filling the calcium-binding site is best modeled as a magnesium ion (Figure 2D). With a RMS deviation of 0.08 Å on the 340 Cα common positions, the two refined structures (Table 1) are almost identical except for the nature of the bound ion, which is either magnesium or calcium. Thus, this ion substitution does not alter the calcium-binding environment and is reversible (Figure 2E). The presence of a single calcium (or magnesium) ion at the top of the heterotrimeric assembly has been proposed to contribute to the stability of the recognition domain of C1q (10).

The X-ray crystal structure of a single-chain version of the homotrimeric globular head of adiponectin had validated the strategy used to generate the recognition domain of this C1q-related protein in a recombinant form (37). The fact that the 3D structure of C1q-scGR is virtually identical to that of the three-chain C1q-derived fragment shows that this strategy can be extended to other proteins containing trimeric gC1q domains.

### Functional Properties of C1q-scGR

**Figure 3:**
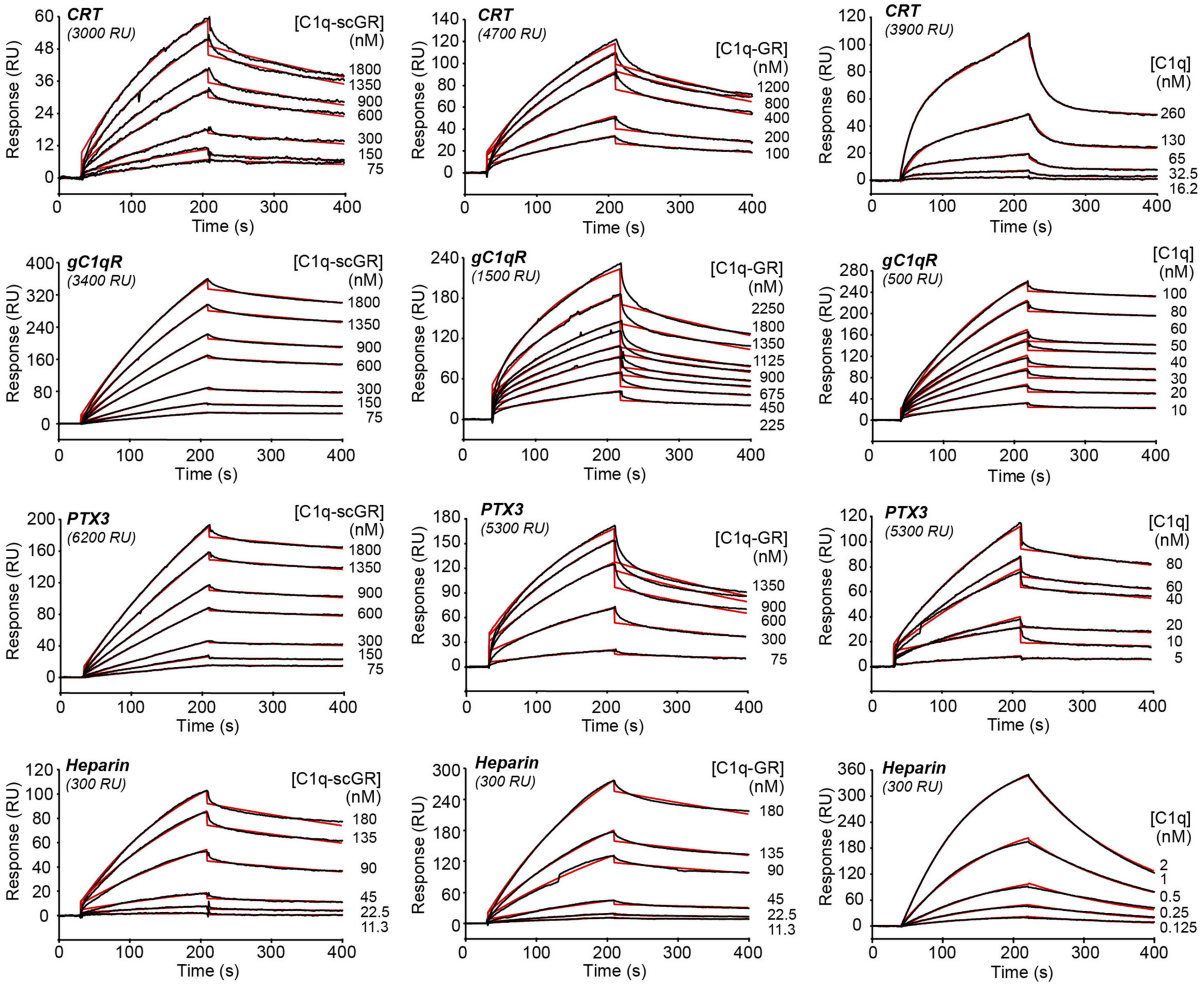
**Kinetic analysis of the interaction of C1q-scGR, C1q-GR, and C1q with immobilized CRT, gC1qR, PTX3, and heparin**. The C1q samples (60 μl) were injected at the indicated concentrations over immobilized CRT, gC1qR, PTX3 in 50 mM Tris–HCl, 150 mM NaCl, 2 mM CaCl_2_, 0.005% surfactant P20, pH 7.4, and over biotinylated heparin in 10 mM Hepes, 150 mM NaCl, 0.005% surfactant P20, pH 7.4. Fits are shown as red lines and were obtained by global fitting of the data using a 1:1 Langmuir binding model, except for the C1q–CRT interaction for which a two-state reaction (conformational change) model was used.

The interaction properties of C1q-scGR were analyzed by surface plasmon resonance (SPR) and compared to those of serum-derived C1q-GR, using known physiological ligands of C1q: the receptor for the globular heads of C1q (gC1qR), CRT, the long pentraxin PTX3, and heparin. All ligands were immobilized on the surface of a sensor chip and the serum-derived and recombinant forms of C1q-GR were used as soluble analytes. Both C1q-scGR and C1q-GR bound to the immobilized ligands with comparable association and dissociation rate constants, yielding comparable apparent *K*_D_ values in the sub-micromolar range (Figure 3; Table 2). This indicated that the recombinant single-chain protein retained the binding capacities of its serum-derived counterpart and was therefore fully functional. The *K*_D_ values obtained here for the interaction with CRT (494–510 nM) and gC1qR (304–344 nM) were in the same range as those reported previously for binding of C1q-GR to CRT (830 nM) (21) and gC1qR (370 nM) (38). The *K*_D_ value obtained for binding to heparin (51 nM) is slightly lower than that obtained previously for the interaction of C1q-GR with 6 kDa heparin (154 nM) (36), a difference that might be explained by the higher molecular weight heparin (15 kDa) used in the present study. Similar results were obtained when the binding experiments were performed in the absence of added calcium in the running buffer (not shown). In light of our structural data, it is likely that the calcium-binding site was still occupied under these conditions, since no chelating agent such as EDTA was used to remove the bound ion.

**Table 2:** **Kinetic and dissociation constants for binding of C1q-scGR, C1q-GR, and C1q to immobilized C1q ligands**.

Soluble C1q sample	Constants	Immobilized C1q ligands			
		CRT	gC1qR	PTX3	Heparin
C1q-scGR	*k*_a_ (M^−1^ s^−1^)	2.80 ± 0.33 × 10^3^	2.22 ± 0.17 × 10^3^	1.32 ± 0.24 × 10^3^	2.51 ± 0.02 × 10^4^
	*k*_d_ (s^−1^)	1.36 ± 0.04 × 10^−3^	6.70 ± 0.31 × 10^−4^	5.52 ± 0.66 × 10^−4^	1.29 ± 0.06 × 10^−3^
	*K*_D_ (nM)	494 ± 47	304 ± 26	444 ± 125	51.5 ± 2.7
C1q-GR	*k*_a_ (M^−1^ s^−1^)	4.63 ± 0.73 × 10^3^	4.40 ± 0.77 × 10^3^	4.59 ± 1.2 × 10^3^	2.09 ± 0.22 × 10^4^
	*k*_d_ (s^−1^)	2.34 ± 0.43 × 10^−3^	1.49 ± 0.20 × 10^−3^	2.17 ± 0.43 × 10^−3^	1.06 ± 0.08 × 10^−3^
	*K*_D_ (nM)	510 ± 88	344 ± 78	599 ± 21	51.5 ± 9.0
C1q	*k*_a_ (M^−1^ s^−1^)		6.80 ± 1.20 × 10^4^	3.87 ± 1.27 × 10^4^	5.49 ± 0.65 × 10^6^
	^a^*k*_a1_ (M^−1^ s^−1^)	7.82 ± 0.12 × 10^3^			
	^a^*k*_a2_ (s^−1^)	4.72 ± 0.90 × 10^−3^			
	*k*_d_ (s^−1^)		4.57 ± 1.3 × 10^−4^	6.72 ± 0.15 × 10^−4^	8.81 ± 1.50 × 10^−3^
	^a^*k*_d1_ (s^−1^)	4.20 ± 0.51 × 10^−2^			
	^a^*k*_d2_ (s^−1^)	4.82 ± 1.12 × 10^−4^			
	*K*_D_ (nM)	590 ± 105	7.20 ± 2.65	18.2 ± 2.6	1.60 ± 0.08

*Values are the means ± SD of at least two separate experiments*.

*^a^The association (*k*_a1_, *k*_a2_) and dissociation (*k*_d1_, *k*_d2_) rate constants of the C1q–CRT interaction were determined by global fitting of the data using a two-state reaction binding model. The resulting dissociation constant was determined from the rate constants: *K*_D_ = 1/[(*k*_a1_/*k*_d1_) (1 + *k*_a2_/*k*_d2_)]*.

When the interaction experiments were performed using full-length C1q as soluble analyte, the binding affinities for all ligands, except CRT, were in the nanomolar range (Figure 3; Table 2). The observation that the C1q globular domain binds to immobilized gC1qR, PTX3, and heparin with a lower affinity (41- to 47-, 24- to 32-, and 32-fold, respectively) than intact C1q is consistent with the fact that C1q-scGR and C1q-GR lack the binding avidity of the hexameric C1q molecule. The decrease in affinity resulted mainly from a decrease in the *k*_a_ value, the *k*_d_ remaining essentially in the same range. A similar 44-fold decreased affinity has been observed previously for the prion protein, another C1q ligand known to be recognized through C1q globular domain (39).

Proper kinetic analysis of the CRT–C1q interaction required the use of a two-state reaction binding model, taking into account conformational changes leading to an increasingly more stable complex formed in two steps, as reported previously for C1q binding to placenta-derived and recombinant CRT (21). The two groups of kinetic constants and the resulting apparent affinity constant (*K*_D_), are listed in Table 2. As proposed previously (21), our data also suggest that CRT recognition by intact C1q implies conformational changes that do not take place in the isolated GR domains. Interestingly, the apparent *K*_D_ values obtained here for the interaction of CRT with C1q or its globular domain were all in the sub-micromolar range, suggesting an interaction mechanism differing from those involving other C1q ligands, such as gC1qR, PTX3, or the prion protein. It should be mentioned that a 2.4- to 7-fold increase in the C1q versus C1q-GR affinity to placenta-derived and recombinant CRT was observed previously, which corresponds anyway to a much lower avidity component compared to the other C1q ligands. Further investigation, including site-directed mutagenesis and/or structural analyses, will be needed to propose a relevant model for the CRT–C1q-GR interaction.

Of note, the same experimental SPR settings did not allow us to compare the binding affinity of C1q and its globular regions for IgG, a classical C1q ligand. Indeed, although full-length C1q readily bound to immobilized human IgG when injected at a concentration of 10 nM, only very weak binding of C1q-GR (either serum-derived or recombinant) injected at concentrations in the micromolar range was observed (Figure S1 in Supplementary Material). This precluded determination of kinetic constants for C1q-GR binding to IgG, but revealed, here too, a difference between full-length C1q and its recognition domains. It is well known that, under physiological conditions, efficient complement activation is triggered by multivalent binding of C1q to antigen–IgG complexes. These conditions can be reproduced artificially in ELISA tests using adsorbed heat-aggregated IgG. Therefore, it is not totally unexpected that the globular regions bind only weakly to immobilized single IgG molecules. The immobilized gC1qR and PTX3 molecules are naturally trimers and octamers, respectively, and it is expected that each of these ligands can bind to a single globular head of C1q, although with less affinity than to full-length C1q.

## Conclusion

The availability of a recombinant functional form of the heterotrimeric globular regions of C1q opens the way for deciphering the molecular basis of the binding versatility of C1q by mapping the residues involved in the recognition of its numerous targets using site-directed mutagenesis. Although it has been shown previously that the three isolated subunits mediate different individual binding properties (12), it is now possible to assess the effects of single residue mutations in the heterotrimeric context of C1q-scGR, as it occurs in native C1q. In addition, given the compact structure of the domain, it appears likely that recognition of certain ligands will involve residues contributed by several subunits (10), a hypothesis that can now be tested experimentally. The availability of two recombinant forms of C1q, the full-length protein (23) and its recognition domain, will allow comparison of their binding and effector properties, taking into account the avidity provided by the hexameric full-length C1q molecule.

In addition to basic research, such artificial gC1q molecules should be of interest for biomedical applications. The contribution of complement in the pathogenesis of many important diseases, including neurodegenerative, infectious, and autoimmune disorders, is now well recognized and C1q is an attractive target for anti-complement therapy (40). C1q-scGR molecules, possibly engineered to form multimers, could thus be used in the fluid phase as natural competitors to inhibit the classical complement pathway at the initial recognition step without any risk of triggering the immune effector mechanisms mediated by the collagen-like regions.

Engineered C1q-scGR molecules might also be used to functionalize magnetic nanobeads or hemoadsorption filters for *in vitro* blood cleansing, as described recently for the carbohydrate recognition domain of human MBL, a recognition protein of the lectin complement pathway (41, 42). MBL-coated devices were shown to efficiently capture bacteria, fungi, and endotoxins from whole human blood and are promising tools in sepsis therapy. Using C1q-scGR would broader the field of application of such devices beyond pathogenic microorganisms because of the capacity of C1q to recognize a variety of potentially noxious altered self elements such as amyloid fibrils and the toxic form of the prion protein. In addition, the fact that C1q specifically reacts with circulating immune complexes and acute-phase proteins such as pentraxins might open the way to the use of C1q-scGR hemoadsorption for the treatment of patients with severe autoimmune diseases such as SLE. It should be mentioned indeed that plasma immunoadsorption on a C1q-column (prepared with immobilized serum-purified porcine C1q) has been successfully used to treat a few SLE patients (43, 44). Using recombinant C1q-scGR, it is now possible to address the potential contamination risks associated with animal serum-derived proteins.

## Author Contributions

NT, CG, and IB designed the study; CM, IB, AC, and NT performed the research; CM, IB, CG, and NT analyzed the data; BB and BG contributed new reagents; NT, CG, and CM wrote the manuscript; all authors revised and approved the final version of the manuscript.

## Conflict of Interest Statement

The authors declare that the research was conducted in the absence of any commercial or financial relationships that could be construed as a potential conflict of interest.

## Footnotes

^1^https://sedfitsedphat.nibib.nih.gov/software

^2^www.bbri.org/RASMB/rasmb.html

## Abbreviations

C1q-GR, globular region of C1q; C1q-scGR, single-chain globular region of C1q; CRT, calreticulin; gC1qR, receptor for the globular head of C1q; SPR, surface plasmon resonance.
