# Supplementary material for: Structural and Functional Characterization of a Single-Chain Form of the Recognition Domain of Complement Protein C1q
Source: Front Immunol. 2016 Mar 2;7:79. doi: 10.3389/fimmu.2016.00079 (PMC4774423; doi:10.3389/fimmu.2016.00079)
Supplement: Supplementary file 1 [file image_1.PDF]

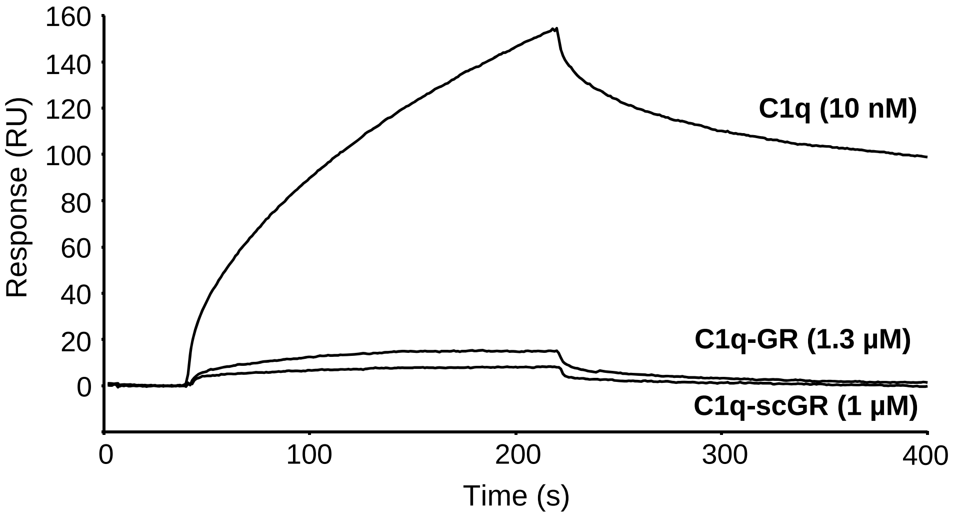

Figure S1: Interaction of C1q-scGR, C1q-GR and C1q with immobilized IgG. Sixty µl of the samples at the indicated concentrations were injected over immobilized human IgG (10650 RU) in 50 mM Tris-HCl, 150 mM NaCl, 2 mM $CaCl_2$, 0.005% surfactant P20, pH 7.4 at a flow rate of 20 µl/min. The specific binding signals shown were obtained by subtracting the background signal over a reference surface with no protein immobilized. The results shown are representative of two independent experiments.
